# The effect of nimodipine on pulmonary function in artificially ventilated patients with aneurysmal subarachnoid hemorrhage

**DOI:** 10.1007/s00701-021-04837-9

**Published:** 2021-04-07

**Authors:** Justyna Lunkiewicz, Giovanna Brandi, Jan Willms, Christian Strässle, Gagan Narula, Emanuela Keller, Carl Muroi

**Affiliations:** Neurocritical Care Unit, University Hospital Zurich, Frauenklinikstrasse 10, 8091 Zurich, Switzerland

**Keywords:** Aneurysmal subarachnoid hemorrhage, Nimodipine, Pulmonary function, Intrapulmonary shunt, Lung injury, Ca-channel blocker

## Abstract

**Background:**

Nimodipine is routinely administered in patients with aneurysmal subarachnoid hemorrhage (aSAH). However, the effect of nimodipine on oxygen exchange in the lungs is insufficiently explored.

**Methods:**

The study explored nimodipine medication in artificially ventilated patients with aSAH. The data collection period was divided into nimodipine-dependent (ND) and nimodipine-independent (NID) periods. Values for arterial partial pressure of oxygen (PaO_2_) and fraction of inspired oxygen (FiO_2_) were collected and compared between the periods. Patients were divided in those with lung injury (LI), defined as median Horowitz index (PaO_2_/FiO_2_) ≤40 kPa (≤300 mmHg), and without and in those with lower respiratory tract infection (LRTI) and without.

**Results:**

A total of 53 out of 150 patients were artificially ventilated, and in 29 patients, the Horowitz index could be compared between ND and NID periods. A linear mixed model showed that during ND period the Horowitz index was 2.3 kPa (95% CI, 1.0–3.5 kPa, *P*<0.001) lower when compared to NID period. The model suggested that in the presence of LI, ND period is associated with a decrease of the index by 2.8 kPa (95% CI, 1.2–4.3 kPa, *P*<0.001). The decrease was more pronounced with LRTI than without: 3.4 kPa (95% CI, 0.8–6.1 kPa) vs. 2.1 kPa (95% CI, 0.7–3.4 kPa), *P*=0.011 and *P*=0.002, respectively.

**Conclusions:**

In patients with LI or LRTI in the context of aSAH, pulmonary function may worsen with nimodipine treatment. The drop of 2 to 3 kPa of the Horowitz index in patients with no lung pathology may not outweigh the benefits of nimodipine. However, in individuals with concomitant lung injury, the effect may be clinically relevant.

## Introduction

Nimodipine is a lipophilic dihydropyridine calcium channel blocker with the ability to cross the blood-brain barrier. It has been shown to reduce the risk of delayed cerebral ischemia (DCI) and subsequently cerebral infarction [1, 4, 15–17], the most important cause of morbidity in patients surviving the initial aneurysmal subarachnoid hemorrhage (aSAH) [9, 16, 18]. So far, nimodipine is the only drug to reduce the incidence of DCI and the risk of poor outcome in an evidence-based manner (class I, level of evidence A) [8, 16] with relative risk calculated as 0.67 (95% CI 0.55 to 0.81) [15]. The compliance with nimodipine was shown to be an independent predictor of better clinical outcome in patients with aSAH [20]. Sandow et al. report that in 28.6% of patients receiving nimodipine as a prophylaxis, the dose had to be reduced and in further 27.7% the drug was discontinued. This was, in 50% of the patients, attributed to hypotension, the well-known and well-described side effect of nimodipine [19]. However, the other causes of drug discontinuation were not mentioned.

There is, however, limited literature that examines disturbances of pulmonary gas exchange during nimodipine therapy. Since over 30 years, calcium channel blockers such as structurally similar to nimodipine, nifedipine, have been considered to have a negative effect on intrapulmonary shunting, decreasing the arterial oxygen partial pressure (PaO_2_) and systemic and pulmonary vascular resistance [6]. However, the effect of nimodipine on oxygen exchange in the lungs is insufficiently explored.

We hypothesize that nimodipine may be associated with increased intrapulmonary shunting and impaired gas exchange which might lead to hypoxemia. This effect may be more pronounced in patients with concomitant lung injury. The purpose of this study is to evaluate the effect of nimodipine on gas exchange and explore its potential mechanisms of impairment.

## Methods and materials

For this exploratory study, data were collected from a consecutive patient series between October 2016 and July 2018 at the Neurocritical Care Unit, University Hospital Zurich, Switzerland. This was a retrospective analysis of data from a prospective study.

**Fig. 1:**
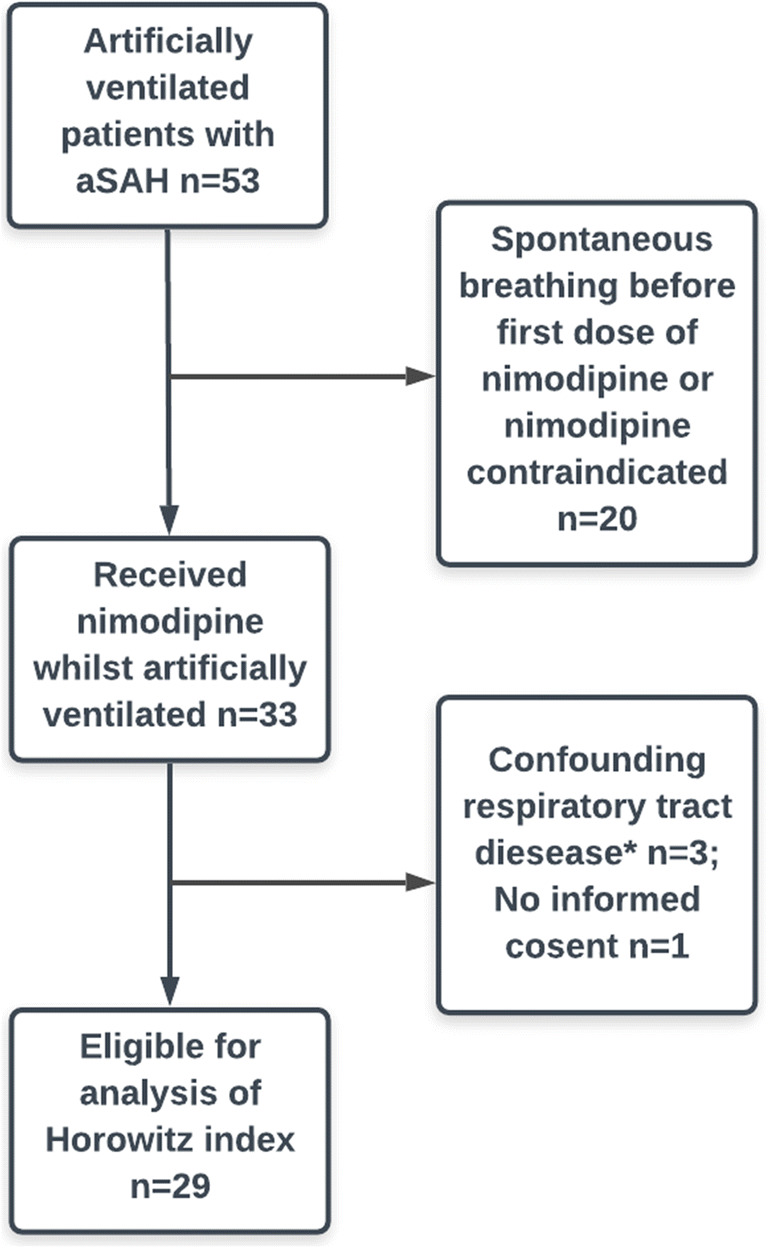
Patient selection based on in- and exclusion criteria as explained in the material and methods. *sleep apnea *n*=2, chronic obstructive lung disease *n*=1

Patients were included if they (1) had radiologically confirmed aSAH, (2) were intubated and ventilated at some point over a continuous period of 24 h, (3) received at least a single-dose nimodipine while intubated, (4) had no known airway pathology likely to be a strong confounder, and (5) informed consent was obtained. The patient selection process is shown in Fig. 1. This explorative study, part of the project “ICU Cockpit,” was approved by the local ethics committee “Kantonale Ethikkommission Zurich.” Written informed consent was obtained from all patients or legal representative respectively

All patients were managed according to a standardized treatment protocol in line with the recommendations of the Neurocritical Care Society’s Multidisciplinary Consensus Conference [8, 13]. The protocol includes nimodipine, if there is no contraindication [3]. We have included patients treated with at least a single dose of nimodipine administered either orally or intravenously at a dosage of 30–60 mg six times daily or 2 mg/h continuously, respectively. In case of significant hypotension, catecholamines were administered in order to maintain an adequate cerebral perfusion pressure. If the dose of noradrenaline had to be escalated to 10 μg/kg/min or higher, the nimodipine dosage was reduced to 50% or, if necessary, completely discontinued. Patients’ baseline demographic and clinical characteristics such as age, gender, aneurysm location, size, Fisher’s grade, Hunt and Hess grade, World Federation of Neurosurgical Societies, and length of stay in intensive care unit were collected. The clinical outcome at time of discharge was assessed according to the Glasgow outcome scale (GOS).

### Data collection

Artificial ventilation was carried out with a use of Hamilton ventilator (Hamilton-G5/S1, Bonaduz, Switzerland). Parameters from the ventilator were streamed to the CNS 220 patient monitor (Moberg Research Inc., Ambler, PA, USA) and forwarded to the “ICU Cockpit,” an IT platform for bio-signal monitoring data consolidation and processing. Parameters, such as fraction of inspired oxygen (FiO_2_), respiratory rate, and positive end-expiratory pressure (PEEP), were continuously recorded with a frequency of 1 Hz. Following blood sampling from an arterial line, arterial blood gas (ABG) analysis (Radiometer, Krefeld, Germany) including arterial partial pressure of oxygen (PaO_2_) were performed routinely every 2 h or more frequently by respiratory worsening. This explanatory study, part of the project “ICU Cockpit,” was approved by the local ethics committee.

### Data analysis

The data collection period was divided into nimodipine-dependent (ND) and nimodipine-independent (NID) periods. ND period, when orally administered, was defined as the time between 1 and 4 h after administration of 30 mg or 60 mg of nimodipine via nasogastric tube. When intravenously administered, ND time was assumed between 30 and 60 min after continuous administration of a maximum rate at 2 mg/h. NID time was defined as the time before first ever dose of nimodipine or was counted from 6 h after the last oral or intravenous nimodipine dose administration irrespective if reduced dosing was required due to hemodynamic instability. The data during the in-between period were discarded in order to obtain clear, not overlapping results between ND and NID periods. A single patient could fluctuate between ND and NID groups (total periods number 66 and 77 for ND and NID, respectively). To identify patients with poor lung function, we used the Horowitz index, defined as the ratio of PaO_2_ and FiO_2_. Subsequently, patients were divided into lung injury (LI) group, defined as median PaO_2_/FiO_2_ ≤40 kPa (≤300 mmHg), and without LI group, with median PaO_2_/FiO_2_ >40 kPa (>300 mmHg). ND and NID periods were compared between patients with and without LI. Further, patients were divided in those with a documented diagnosis of a lower respiratory tract infection (LRTI). The diagnosis was based on the definitions of American Thoracic Society and current literature [11]. As most patients received treatment with antibiotics for at least 7 days, the duration of LRTI was defined as 7 days unless longer periods were documented. ND and NID periods were compared between patients with and without LI.

### Statistical analysis

Statistical analysis was performed using SPSS (SPSS Ver. 25, IBM, Armonk, NY, USA). The large datasets were analyzed using Python 3.8.2 in conjunction with Pandas 1.2.1 and NumPy 1.20.0 before statistical analysis.

In brief, median values of variables of interest were compared by the Wilcoxon signed-ranks test. Binominal variables between groups were compared by the Fisher’s exact test. However, as the current longitudinal dataset was unbalanced, i.e., showed different numbers of observations per subject and/or subjects measured at different time points, a linear mixed model analysis was performed [21]. Continuous values are given as median and interquartile range (IQR) or mean ±standard deviation (SD). A *P*-value <0.05 was considered as statistically significant.

## Results

Based on our inclusion criteria, a total of 29 were eligible for analysis (Fig. 1). Baseline characteristics are summarized in Table 1. The majority of patients presented with a clinically severe hemorrhage and had an unfavorable outcome (GOS 1–3). Overall, median Horowitz index (PaO_2_/FiO_2_) during ND and NID periods did not show any significant differences (Table 2). Comparison of potential confounding factors such as mean arterial pressure and partial pressure of arterial carbon dioxide (PaCO_2_) showed no difference between ND and NID groups (Table 2). Decrease in PaO_2_/FiO_2_ during ND periods was observed in 50% of patients with signs of LI and in 27% of patients without LI. However, the difference was statistically not significant (*P*=0.273, Fisher’s exact test).

**Table 1:** Patients’ baseline characteristics

Gender		
Male, *n* %	7	(24%)
Female, *n* %	22	(76%)
Age, mean ±SD (years)	56.9	± 12.2
WFNS		
Grades 1–3, *n* %	12	(41%)
Grades 4–5, *n* %	17	(59%)
Aneurysm location		
Anterior circulation	23	(79%)
Posterior circulation	6	(21%)
Length of ICU stay, mean ±SD (days)	27.6	± 18.8
GOS		
GOS 4–5, *n* %	4	(14%)
GOS 1–3, *n* %	25	(86%)

*GOS* Glasgow outcome scale, *ICU* intensive care unit, *SD* standard deviation, *WFNS* World Federation of Neurosurgical Societies

**Table 2:** Comparison of median values between nimodipine dependent and independent periods

	ND, median (IQR)	NID, median (IQR)	Sig.
Exposure time, hrs	33 (5–109)	100 (59–272)	*N/A*
All patients, *n*=29 PaCO_2_, kPa	5.3 (4.9–5.6)	5.2 (5.03–5.5)	*P=0.469*
All patients, *n*=24* Mean arterial pressure	101.4 (94.2–110.5)	106.7 (99.6–112.8)	*P=0.274*
All patients, *n*=29			
FiO_2_, %	38.0 (33.3–41.0)	39.0 (35.0–42.0)	*P*=0.718
PEEP, cmH_2_O	6.6 (6.0–10.0)	6.9 (6.0–12.0)	*P*=0.202
Horowitz index, kPa	40.0 (32.4–45.8)	38.2 (33.0–44.0)	*P*=0.973
LI, *n*=18			
FiO_2_, %	40.0 (35.8–45.1)	40.0 (39.0–45.8)	*P*=0.671
PEEP, cmH_2_O	10.0 (7.6–11.0)	10.0 (6.5–13.0)	*P*=0.385
Horowitz index, kPa	33.5 (28.3–40.2)	35.1 (31.7–38.2)	*P*=0.640
No LI, *n*=11			
FiO_2_, %	35.0 (30.5–38.0)	35.0 (31.0–36.0)	*P*=0.883
PEEP, cmH_2_O	6.0 (6.0–6.1)	6.0 (6.0–6.2)	*P*=0.750
Horowitz index, kPa	48.8 (40.3–52.3)	49.3 (41.0–51.3)	*P*=0.557

*hrs* hours, *IQR* indicates interquartile range, *LI* lung injury, *N/A* not applicable, *ND* nimodipine dependent, *NID* nimodipine independent, *PaCO*_*2*_ partial pressure of arterial carbon dioxide, *sig* significance; Wilcoxon-signed ranks test; *missing data in database

**Fig. 2:**
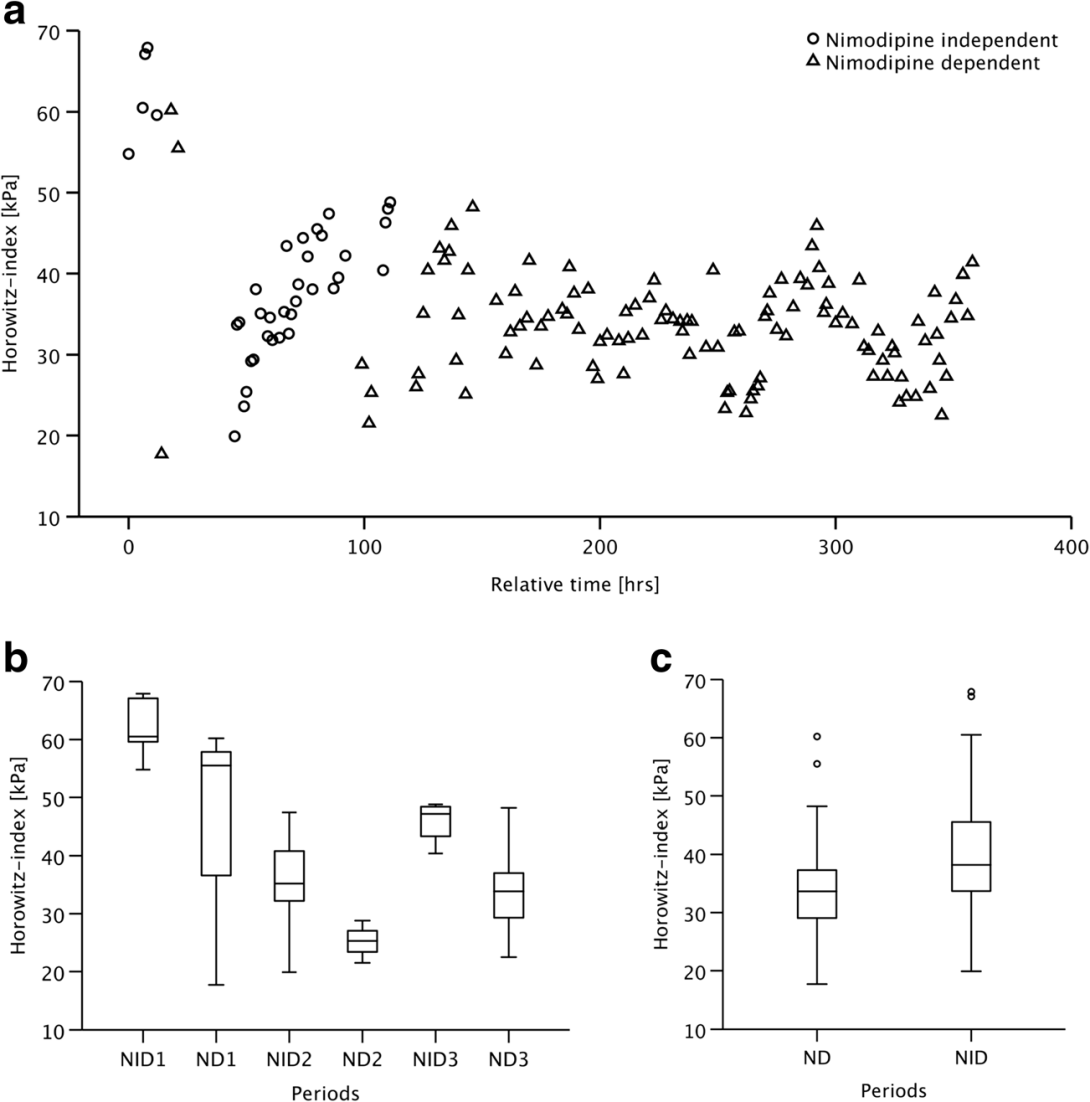
**a** An illustrative case of an individual patient’s response to nimodipine treatment measured as Horowitz index in the time course of treatment. **b** As nimodipine treatment was withheld and restarted multiple times, nimodipine-independent (NID) and nimodipine-dependent (ND) periods are numbered accordingly. Values are shown as box plots**. c** Values over the whole observation period are shown as box plots, stratified by ND and NID. The graphs show the difficulty in the analysis of unbalanced longitudinal datasets

Analyzing individual patients more closely, a decrease in the Horowitz index during ND periods was noted in some patients, while nimodipine treatment was withheld and restarted multiple times (Fig. 2a and b). However, this aspect was seemingly lost when the corresponding periods were clustered together and the Horowitz indices averaged (Fig. 2c).

The linear mixed model analysis showed that nimodipine has an effect on the Horowitz index. During ND period, the Horowitz index was 2.3 kPa (95% CI 1.0 to 3.5 kPa, *P*<0.001) lower when compared to NID period. The model suggests that in the presence of lung injury (LI), ND period is associated with a decrease of the Horowitz index by 2.8 kPa (95% CI 1.2 to 4.3 kPa) (*P*<0.001) (Table 3). If LI, however, is not present, Horowitz index did not change between ND and NID periods in a statistically significant way. The analysis of the data using the variable “lower respiratory tract infection (LRTI)” instead of “LI” showed that ND periods were associated with a decreased Horowitz index with and without the presence of LRTI. The decrease was more pronounced with LRTI than without: 3.4 kPa (95% CI 0.8 to 6.1 kPa) vs. 2.1 kPa (95% CI 0.7 to 3.4 kPa), *P*=0.011 and *P*=0.002, respectively (Table 3).

**Table 3:** Linear mixed model analysis estimates of fixed effects^a^

						95% CI	
	Estimate	SE	df	*t*	Sig.	Lower	Upper
Model: Horowitz index by nimodipine with time							
Intercept	39.2	1.6	28.5	23.8	*P*<0.000	35.9	42.6
ND	−2.3	0.6	1011.1	−3.5	*P*<0.000	−3.5	−1.0
NID	0^b^	0	.	.	.	.	.
Model: Horowitz index by nimodipine, LI with time							
Intercept	34.5	1.3	25.1	26.0	*P*<0.000	31.8	37.2
ND * no LI	12.0	2.3	30.1	5.3	*P*<0.000	7.4	16.7
NID * no LI	13.6	2.3	29.4	6.1	*P*<0.000	9.0	18.2
ND * LI	-2.8	0.8	901.4	−3.6	*P*<0.000	−4.3	−1.3
NID * LI	0^b^	0	.	.	.	.	.
Model: Horowitz index by nimodipine, LTRI with time (1)							
Intercept	38.5	1.8	40.0	21.3	*P*<0.000	34.8	42.1
ND * no LRTI	−1.2	1.0	738.4	−1.2	*P*=0.236	−3.1	0.8
NID * no LRTI	0.9	0.9	674.3	1.0	*P*=0.316	−0.9	2.6
ND * LTRI	−3.4	1.4	787.60	−2.5	*P*=0.011	−6.1	−0.8
NID * LRTI	0^b^	0	.	.	.	.	.
Model: Horowitz index by nimodipine, LTRI with time (2)							
Intercept	39.4	1.7	29.0	23.7	*P*<0.000	36.0	42.8
ND * LRTI	−4.3	1.3	817.4	−3.4	*P*=0.001	−6.8	−1.8
NID * LRTI	−0.9	0.9	674.3	−1.0	*P*=0.316	−2.6	0.9
ND * no LRTI	−2.1	0.7	1055.3	−3.1	*P*=0.002	−3.4	−0.7
NID * no LRTI	0^b^	0	.	.	.	.	.

^a^Dependent variable: Horowitz index (kPa)

^b^This parameter is set to zero because it is redundant. *SE* standard error, *df* degree of freedom, *t t*-test, *sig.* significance, *CI* confidence interval

## Discussion

Our data suggest that in patients with LI or during periods with LRTI, pulmonary function may worsen with nimodipine treatment. Based on the modeling, the decrease of the Horowitz index was 2.5 to 3.6 kPa and can be clinically relevant if already respiratory partial insufficiency is present.

While analyzing our datasets, we have taken under the consideration the following aspects: multiple samples from one patient taken over long time, changes in patient’s condition over time, the fluctuation of an individual patient between ND and NID periods, the variation of the number of such periods per patient, and the variation of the number of measurements per period. To show the effect, one needs to perform analysis that recognizes time-invariant and time-varying covariates. Linear mixed model analysis allowed for this and furthermore was able to accommodate all of the data that are available for a given subject, without dropping any of the data collected for the subject, under mild assumptions regarding the missing data. Therefore, this effect could only be detected by a linear mixed model analysis and a simplification by averaging the repeated measurements led to a loss of precious information.

Our results are in keeping with few case reports describing hypoxic episodes following nimodipine administration and further literature on hypoxemia as a known complication of medication with vasodilating properties, particularly when concomitant pulmonary disease is present [2].

Devlin et al. report a case following traumatic SAH who suffered acute life-threatening hypoxemia following two separate administrations of single dose of nimodipine on background of diagnosis with adult respiratory distress syndrome (ARDS). The authors hypothesized that in patients of ARDS, nimodipine, by vasodilating the pulmonary artery and potentially diminishing reflex hypoxic pulmonary vasoconstriction, contributes to increased ventilation/perfusion ratio mismatch and severe hypoxia [7]. In a further case report describing nimodipine induced hypoxemia, a patient with aSAH following initiation of nimodipine therapy developed respiratory distress on multiple occasions requiring reintubation. The authors concluded that hypoxemia episodes were most likely caused by the drug and speculated that responsible mechanism was based on greater amounts of systemic venous blood passing through hypoventilated or nonventilated lung areas leading to pulmonary shunting [2]. Furthermore, Kieninger et al. reported a brief impairment of the pulmonary gas exchange on the second day in comparison to the first day of the long-term continuous intra-arterial nimodipine infusion in patients with severe refractory cerebral vasospasm after SAH [12]. Gerloni et al. presented a case report on hypoxemia and hypotension induced by accidental nimodipine overdose that supported the hypothesis suggested by Develin et al. [7, 10].

However, Bolt et al. investigated the effect of calcium channel blockers including nimodipine on pulmonary shunting in anesthetized patients scheduled for aortocoronary bypass surgery. The study showed that nimodipine leads to decline in pulmonary vascular resistance, almost no change in pulmonary shunting, a rise in PaO_2_ as well as in cardiac output [5]. Bolt et al. found no change in pulmonary shunting but only investigated patients without respiratory dysfunction. Our data are in keeping with the conclusion made by Bolt et al. that in patients with normal lung function nimodipine has no major impact on hypoxia and may suggests potential mechanism of action of nimodipine on pulmonary function. Nimodipine while increasing cardiac output might lead to preferential pulmonary vascular recruitment in non-ventilated areas of the lung and promote shunting [5, 14]. Hypoventilated lung regions divert blood to better ventilated areas as part of autoregulatory hypoxic pulmonary vasoconstriction response optimizing ventilation/perfusion ratios. In diseased lungs, this mechanism plays a pivotal role in maintaining adequate PaO_2_ [5]. This mechanism may be impaired by nimodipine by reducing pulmonary vascular resistance also in pathological lung regions and increasing the pulmonary blood flow to the non-ventilated lung areas, resulting in increased pulmonary shunting. To note, according to US Food and Drug Administration-approved labeling text for oral use of Nimotop (nimodipine), there were no pulmonary adverse effects reported [3].

There are few study limitations that need to be considered when interpreting our results. This is a retrospective study, and arterial blood gas sampling were performed based on clinical need rather than at higher frequency for research purposes. Any signs suggesting deterioration in pulmonary function based on clinical observation and monitoring would have prompted nursing and medical stuff to implement the measures to improve oxygenation of the patient and later on perform arterial blood gas analysis potentially after the oxygenation improving measures took an effect. Furthermore, nimodipine administration was withheld if noradrenaline requirements rose above 10 μg/min. This often occurred when the patient suffered from a concomitant infection. In those cases, data would be analyzed as nimodipine independent after discarding data from the overlapping period. Systemic inflammatory and vasodilating response or vasoactive drugs themselves could have been concomitant factors affecting pulmonary function. Moreover, patients whose pulmonary function significantly worsened due to nimodipine would have had nimodipine withheld and subsequently providing little samples whereas those who tolerated nimodipine well would continue to have nimodipine administered and provide multiple ABG measurements. This could have contributed to the weaker detection of the negative effect of nimodipine on pulmonary function.

Shunt fraction, as defined by the equation Shunt=Q_s_/Q_t_= (CcO_2_ – CaO_2_)/(CcO_2_ – CvO_2_), where Q_s_ = Pulmonary Physiologic Shunt (mL/min); Q_t_ = Cardiac Output (mL/min); CcO_2_ = End-pulmonary-capillary Oxygen Content; CaO_2_ = Arterial oxygen content; and CvO_2_ = Mixed Venous Oxygen Content, requires mixed venous blood measured by pulmonary catheter. Further studies to explore the effect of nimodipine on pulmonary function mechanism should be prospective to enable estimation of the shunt fraction either by blood sampling from pulmonary catheter or by other methods.

## Conclusion

In conclusion, our data suggest that in patients with LI, or during periods with LRTI, pulmonary function may worsen more with nimodipine treatment. The drop of 2–3 kPa of the Horowitz index in patients with no lung pathology may not outweigh the benefits of nimodipine. However, in individuals with concomitant LI, the effect may be more clinically relevant and prompts further consideration. Further prospective studies are needed to describe the mechanism of an effect of nimodipine on lung function in patients with and without lung pathology.
